# Prevalence of traumatic brain injury among the guests at a low-barrier homeless shelter

**DOI:** 10.1186/s13104-021-05452-8

**Published:** 2021-02-04

**Authors:** Nicholas Benjamin Ang, Jason Adam Wasserman

**Affiliations:** 1 Oakland University William Beaumont School of Medicine, Rochester Hills, MI USA; 2 Department of Foundational Medical Studies and Department of Pediatrics, Oakland University William Beaumont School of Medicine, Rochester Hills, MI USA

## Abstract

**Objective:**

This study aimed at determining the prevalence of traumatic brain injuries (TBI) among guests staying at a low-barrier homeless shelter who represent an especially vulnerable subset of individuals experiencing homelessness.

**Results:**

A total of 21 out of 35 shelter guests participated in the survey. We found that 17 (81.0%) had experienced at least one traumatic brain injury in their lifetime and 15 (71.3%) had TBI associated with loss of consciousness. In addition, 7 (33.3%) of the participants had experienced TBIs rated as moderate to severe. Of the participants with head trauma history, 16 (94.1%) experienced their injury before their first onset of homelessness. Compared to both the general population and the broader population of individuals experiencing homelessness, those in this sample were significantly more likely to experience TBI (95% CI 0.0000:0.2857; p < 0.001 and 95% CI 0.3333:0.7619; p < 0.015, respectively) and significantly more likely to experience severe TBI (95% CI 0.0000:0.09524; p < 0.001).

## Introduction

Traumatic Brain Injury (TBI) is defined by the CDC as “a blow or jolt to the head or a penetrating head injury that disrupts the normal function of the brain” [1]. With automobile accident, athletic injury, and assault among its most common causes [2], TBI is a highly prevalent and serious injury with multifactorial consequences impacting physical, cognitive, and social function. According to the World Health Organization, around 30–40% of all injury-related deaths world-wide are a result of a TBI [1]. Furthermore, neurologic injuries currently represent the leading cause of neurological disability, ahead of diseases such as Alzheimer’s and cerebrovascular disorders [1].

There is significant variation in the definition and measurement of TBI, rendering comparisons of prevalence between populations difficult. Reported rates vary widely across different studies. One study, for example, reports approximately 50–60 million people experience a first incident every year [3], with some estimates showing a lifetime prevalence of TBI totaling nearly half the world’s population [3]. Another study reported 12% of the general population has experienced a TBI with a loss of consciousness [4]. A separate study in Ohio also measured TBI’s with a loss of consciousness and found 21.7% of people in the general population incurred a TBI of any severity, and 2.6% have experienced a moderate to severe TBI with loss of consciousness [5].

One thing is particularly clear from the literature: TBI does not affect all populations equally. People experiencing homelessness are at especially high risk of TBI of all severities [6–11]. One meta-analysis of 22 studies shows an average of 53.1% of individuals experiencing homelessness had at least one TBI of any severity, and 22.5% have had at least one moderate to severe TBI [10]. This study suggests that the rate of overall TBI’s among individuals experiencing homelessness is 2.5 to 5 times greater than the general population, and that risk is 10 times greater for moderate to severe TBI’s [6, 8–10]. These data are not surprising when one considers that persons experiencing homelessness are vulnerable in numerous ways. For example, experiencing homelessness places one at a higher risk of physical or sexual assault [12].

The neuro-cognitive impairments associated with TBI are particularly challenging to address. These impacts may not appear until years later and they can permanently change the victim’s mental and cognitive health if left untreated. As a result of reduced access to treatment, education, and opportunity, individuals experiencing homelessness may be at an even greater risk for sustaining the negative effects of TBI’s. In turn, TBI’s may be a contributing factor to the high rates of cognitive dysfunction found among those who have experienced homelessness compared to the general population [7], including problems with impulse control, goal-setting, and memory, which may exacerbate the challenges of matriculating from service programs into housing.

An additional complexity concerns whether TBI represents a causal factor that promotes homelessness, or an effect of the risk exposure associated with homelessness. In support of the former, one study showed approximately 70% of individuals experiencing homelessness had at least one TBI before the onset of their homelessness [11]. People who have sustained severe TBI’s have a harder time with social integration and facial affect recognition [13]. The authors suggest that TBI’s may increase one’s difficulty navigating society and maintaining various aspects of their lives, potentially increasing the risk for chronic homelessness. On the other hand, another study found that the longer a person was homeless, the more likely they were to have sustained a TBI [14]. In other words, the longer someone experiences homelessness, the more likely the chance of experiencing a TBI incident. Ultimately, previous research indicates that TBI’s can be both a cause and a result of homelessness [6].

Limited understanding of the prevalence, causes, and effects of TBI on homelessness warrants additional research that can inform better interventions. Adding to this complexity is the fact that homelessness itself represents a heterogenous population who face different levels of risk. Short term homelessness, for example, likely carries less risk than chronic homelessness; those living on the street are at greater risk than those “doubling up” with friends or family. People staying at low-barrier shelters represent a more vulnerable subset within the overall population of individuals experiencing homelessness. A low-barrier shelter is defined as a form of emergency shelter for individuals experiencing homelessness which facilitates easy access by keeping historical barriers to shelter entry to a minimum [15]. This includes a variety of entry requirements that otherwise limit who can enter the shelter, when, and how often, including sobriety requirements. This subgroup may exhibit particularly problematic cognitive deficits and behaviors associated with TBI, and may at the same time be at greater risk of a TBI event such as likelihood of being assaulted. Yet no research to date has evaluated this particularly vulnerable subset of individuals experiencing homelessness.

This study reports data on the prevalence of TBI among a clients of a low-barrier homeless shelter as well as investigating whether a TBI event preceded matriculation into homelessness.

## Methods

The participants in this study were guests at a low-barrier homeless shelter for adults in a mid-sized city located within the metropolitan statistical area of a large urban city in the Midwest United States. All shelter guests were invited to participate via an announcement by the shelter staff about the study. Ultimately, 21 out of the 35 guests completed the survey. All interviews were conducted remotely via video conferencing due to social distancing mandates associated with COVID-19. Informed consent was obtained by reading a consent form over the video conference and no compensation was offered to participants. The interviews were conducted by a second-year medical student who was trained by the faculty researcher in the administration of the Ohio State TBI Identification Method (OSU TBI-ID) [16], as well as other relevant interviewing best practices.

Data on participant age, gender, race, and education were collected, as well information about the length of their current episode of homelessness, time of first homeless episode, and frequency of homelessness in the past 3 years [17]. The OSU TBI-ID [16] was used to measure traumatic brain injury events, including overall prevalence of TBI and whether there was loss of consciousness. The severity of each incident was determined using standardized criteria and rated as mild, moderate, or severe [9]. A single sample difference of proportions test using the binomial distribution was used to compare prevalence of TBI with parameters derived from existing literature on TBI prevalence among both the general population and the general population of individuals experiencing homelessness [3, 5, 6, 8, 10]. The rate of TBI with loss of consciousness was used as the comparison criteria for the general population. In order to compare individuals experiencing homelessness staying at a low-barrier shelter to the general population of those experiencing homelessness, the prevalence of TBI regardless of loss of consciousness was used as the comparison criterion. Two different measurements were used in order to test against the most reliable parameter estimates for each comparison population.

## Results

Out of the 21 participants in this study, 90.5% were male (n = 19) and 9.5% were female (n = 2). 61.9% of the participants identified as black [13] and 38.1% as white [8]. The average age was 50.4 years old (SD = 9.77).

Overall, we found that 80.9% of the participants had experienced some degree of TBI ranging from mild to severe in their lifetime and 71.4% of the participants have experienced a TBI with loss of consciousness. The general population has a 8–12% rate of TBI with loss of consciousness [8, 9]. Compared to the upper limit of 12% among the general population, people experiencing homelessness staying at a low-barrier shelter (71.4%) have a significantly greater rate of TBI with loss of consciousness (95% CI 0.0000:0.2857; p < 0.001). In terms of severity, we found that 47.6% of the participants have experienced a mild TBI, 19.0% have experienced a moderate TBI, and 14.3% have experienced a severe TBI. The general population has a 2.6% rate of moderate to severe TBI [5]. Comparing this value to the combined 33.3% rate of moderate to severe TBI in the data we collected, this more vulnerable subgroup of individuals experiencing homelessness has a significantly greater chance of experiencing worse TBI’s (95% CI 0.0000:0.09524; p < 0.001). In addition, 41.9% of the participants have had 1–2 TBI incidents in their lifetime while 38.1% have had 3 or more. This data shows just how prevalent and serious TBI’s are within this sample population of individuals experiencing homelessness.

We also compared our sample population to the general population of individuals experiencing homelessness. The general population of individuals experiencing homelessness has a 53.1% rate of TBI regardless of loss of consciousness [6, 11]. We found that people experiencing homelessness who are staying at a low-barrier shelter have a TBI rate of 81.0% and are significantly more likely to experience TBI regardless of loss of consciousness (95% CI 0.3333:0.7619; p < 0.015).

Regarding temporal occurrence of TBI’s in relation to the first incident of homelessness, we found that 94.1% of the participants with a history of TBI experienced their first TBI *before* they experienced homelessness. This complements previous data suggesting TBI often precedes homelessness [11] and may be implicated as a cause of homelessness, not just an exposure risk associated with experiencing homelessness.

## Discussion

While previous research had established that rates of TBI among persons experiencing homelessness were disproportionately high, this appears to be even more true for the particular population utilizing a low-barrier homeless shelter. This suggests that those persons experiencing homelessness who are more likely to be chronically homeless and to have greater rates of mental illness and addiction, are also much more likely to have TBI compared to the general population. A vast majority of participants had experienced their first TBI before their matriculation into homelessness. This, combined with the fact that rates of TBI among this subpopulation are higher compared to the broader population of people experiencing homelessness, suggests that TBI represents both a cause and an effect of homelessness. This particularly vulnerable subset of individuals experiencing homelessness face more challenges with respect to matriculating to housing and are also more exposed to the risks associated with homelessness such as assault by virtue of spending more time on the street. Without the proper treatment, these negative effects may have lasting implications that perpetuate their homelessness.

People experiencing homelessness, especially the most vulnerable among them, require increased support for the associated symptoms and long-term effects of TBI. There should be greater access to TBI screening and treatment, with programs specifically targeting those who are experiencing homelessness, and perhaps especially those who utilize low-barrier and emergency services or sleep on the street itself. This is especially important because early intervention can help mitigate long term side effects, and the consequences of TBI (e.g. problems with memory, goal-setting, and impulse control) may be factors that make it challenging to exit homelessness. Finally, educating homeless services providers on how to design programs that are cognizant of people living with TBI will create a more useful support structure for these individuals.

Traumatic brain injuries are a pressing issue in healthcare and come with many long term neurological and social effects. Data collected across the world in previous studies and data from this study demonstrates that those experiencing homelessness are disproportionately affected by TBI’s. This study shows that a subpopulation of individuals experiencing homelessness staying at a low-barrier shelter are more vulnerable to TBI’s. It shows that there is a clear need for improved TBI treatment and opportunity for these individuals to close this gap in health equity. Future studies should examine additional correlates of TBI among populations of people experiencing homelessness, as well as ways to directly measure the causal relationship between TBI and homelessness. Furthermore, future research should expand upon this project by collecting more data on other vulnerable subgroups of individuals experiencing homelessness.

## Limitations

Due to the relatively small number of people staying at the low-barrier shelter, this study had a modest sample size (n = 21). Additionally, while this data gives a glimpse at the prevalence of TBI’s in a specific area, it may not be completely representative of the general population. Since the data was only collected at one shelter, this data is limited to the population staying at that shelter. Furthermore, people who have experienced TBI may have been less likely to participate in this study, which was conducted virtually, due to difficulty looking at screens, though this would theoretically mean the actual rate of TBI in the population is even higher than we report here. Finally, while the OSU TBI-ID is a validated instrument, there remain potential limitations to any measures of TBI that rely on participant self-report.

## Data Availability

The data and study protocol are available from the corresponding author upon request.

## Abbreviations

TBI: Traumatic brain injury
CDC: Center for Disease Control
COVID-19: Corona Virus Disease 19’
